# FLT3LG and IFITM3P6 consolidate T cell activity in the bone marrow microenvironment and are prognostic factors in acute myelocytic leukemia

**DOI:** 10.3389/fimmu.2022.980911

**Published:** 2022-08-23

**Authors:** Haiyan Chen, Meng Wu, Hongping Xia, Songjie Du, Guoren Zhou, Guangfeng Long, Yanan Zhu, Xu Huang, Daheng Yang

**Affiliations:** ^1^ Institute for Cancer Research, School of Basic Medical Science of Xi’an Jiaotong University, Xi’an, China; ^2^ Department of Clinical Laboratory, Children’s Hospital of Nanjing Medical University, Nanjing, China; ^3^ Department of Pathology, School of Basic Medical Sciences & Key Laboratory of Antibody Technique of National Health Commission, Nanjing Medical University, Nanjing, China; ^4^ Department of Medical Genetics, Institute of Basic Medical Sciences, Chinese Academy of Medical Sciences & Peking Union Medical College, Beijing, China; ^5^ Jiangsu Cancer Hospital & The Affifiliated Cancer Hospital of Nanjing Medical University & Jiangsu Institute of Cancer Research, Nanjing, China; ^6^ Translational Medicine Institute, Xi’an Jiaotong University Health Science Center, Xi’an, China

**Keywords:** acute myelocytic leukemia, bone marrow microenvironment, dendritic cell activation, T cell activation, FLT3LG, IFITM3P6

## Abstract

Acute myelocytic leukemia (AML) is a malignancy of the stem cell precursors of the myeloid lineage. CD4+ and CD8+ T cells play pivotal roles in influencing AML progression but are functionally suppressed in the bone marrow microenvironment. We aimed to find hub genes related to T cell exhaustion and suppression, thereby providing evidence for immunotherapy. In this study, gene transcriptome expression data from TCGA and TARGET databases were utilized to find key genes. Firstly, CIBERSORT immune cell infiltration algorithm and WGCNA method were used to identify CD4+ and CD8+ T cells-related genes. Univariate and multivariate cox regression analyses were then introduced to construct the overall survival prognosis model and included hub genes. The ESTIMATE and ssGSEA scoring methods were used to analyze the correlation between the hub genes and immune activity. Single-cell transcriptome analysis was applied to detect the immune cells expressing hub genes, hence, to detect exact mechanisms. Consequently, FLT3LG and IFITM3P6 were determined to be positively correlated with patients’ overall survival and microenvironment immune activity. Further study suggested FLT3-FLT3LG and IFITM3P6-miR-6748-3p-CBX7 signaling axes were involved in CD4+ and CD8+ T cells activation. This may be one of the mechanisms of T cells suppression in AML.

## Introduction

Acute myelocytic leukemia (AML) is a group of malignant clonal diseases that originate from the myeloid stem and progenitor cells and are highly heterogeneous in immunophenotype, cytogenetics, and molecular genetics (1). It’s the most common acute leukemia in adults (~80% in this group), and the proportion of pediatric patients is about 20% (2). Similar to other malignant tumors, genetic variations consist of the main reason that leads to neoplastic changes and clonal proliferation. The incidence of AML elevated with age, from ~1.3 cases per 100 thousand population less than 65 years old, to 12.2 per 100 thousand population that over 65 years old (3). With a very variable prognosis and a high mortality rate, 5-year overall survival in AML cases is less than 50%, and 20% of elderly patients will survive 2 years after diagnosis (4, 5). In the past decades, treatment paradigms were still unchanged with survival curves remaining stagnant (6).

The current regimens include a high-intensity induction phase wherein cytotoxic chemotherapy, or other target-specific agents are administered based on disease profile as well as patient risk and comorbidities (6, 7). Due to the minimal residual disease in the bone marrow (BM) microenvironment, failure in treatment often occurred (8, 9). Therefore, a better understanding of the AML microenvironment is crucial for preventing tumor development and designing an effective treatment regimen. Studies have reported various disorders in T cell immunity, including increased T regulatory cells, decreased T helper cells, exhausted T cells, functional T cell suppression and abnormal activity of transcription factors in the presence of AML (10). Consequently, T cell-associated functions are changed to allow immunity evasion of tumor cells. The importance of T cell in antileukemia is confirmed by Lamble et al., they showed an association between T cells and clinical outcomes based on their research. Patients with higher BM T cell percentages (over 78.5% of total lymphocytes) presented favorable overall survival (11). In addition, an *in vitro* co-culture experiment of T cells and AML leukemic cells revealed that pooled CD4+ and CD8+ cells cytotoxic against blasts (32%, 30:1 E/T ratio) (12). The antitumor effect of CD8+ T cells is mainly attributed to it can recognize AML-derived mutated peptides and induce a cytotoxic effect on tumor cells. One important mechanism was revealed in a study that CD8+ T cells could disrupt AML progression caused by mutation of nucleophosmin 1 (NPM1) or FMS-like tyrosine kinase receptor 3 internal tandem duplication (FLT3-ITD) (13, 14). Other studies demonstrated that CD4+ T cells prompt apoptotic effect on AML cells and are mainly associated with INF-γ release (15).

It has been well documented cytotoxic effects of CD4+ and CD8+ T cells against AML cells, which consists of important mechanisms in influencing disease relapse and drug resistance in the tumor microenvironment (TME) (16, 17), blocking tumor progression, invasiveness, and metastasis, and even maintaining a cancer stem-like phenotype (18). But so far, the molecular and cellular mechanisms of T cell exhaustion and functional suppression remain unclear. A better understanding of these will give a deep insight into the balance of tumor immune surveillance and cancer cell evasion in TME, and help to develop novel immunotherapeutic approaches.

The rapid development of bioinformatics facilitates the exploration of potential mechanisms driving tumor progression. In this study, we sought to determine the relationship between hub genes and T cells in TME to reveal the landscape of AML progression.

## Materials and methods

### Data acquisition and processing

Gene transcriptome expression data of AML were obtained from The Cancer Genome Atlas (TCGA) (https://portal.gdc.cancer.gov/) and Therapeutically Applicable Research to Generate Effective Treatments (TARGET) (https://ocg.cancer.gov/programs/target/data-matrix) databases. The blood gene expression data of healthy controls were obtained from the Genotype-Tissue Expression (GTEx) (https://gtexportal.org/home/datasets) database, including 755 cases. All expression data were raw count values. Before analysis, these values were converted into transcripts per million (TPM) values.

Batch correction of the merged data was performed by the normalizeBetweenArrays function of the *limma* package. Finally, the expression data were filtrated with a criterion by the average expression level of each gene across samples > 0.1, and the repeated genes were averaged.

### Analysis of immune cell infiltration

Firstly, the immune cell infiltration landscape of TCGA and TARGET data was explored to find relationships between infiltrating immune cells, especially CD4+ and CD8+ T cells, and AML patients’ overall survival (OS). A deconvolution algorithm CIBERSORT (https://cibersortx.stanford.edu/) calculates the composition and proportion of infiltrating immune cells (19). It has been widely used to reveal the immune cell subtypes infiltrating numerous cancers (20, 21). With matched R script v1.03 (last updated 07-10-2015) and feature genes expression matrix of 22 immune cell subsets (downloaded from CIBERSORT website) as background expression data, the infiltration features of these immune cells in each sample were determined. Samples with *P* < 0.05 were included for further analysis. The analysis was based on *e1071*, *parallel* and *preprocessCore* packages.

### Screening of immune cell infiltration-related genes

Next, we introduced weighted gene co-expression network analysis (WGCNA), which is used to analyze gene expression patterns of multiple samples, to identify core modules and central genes that are associated with infiltrating CD4+ and CD8+ T cells (22). WGCNA method can cluster genes and form modules by similar gene expression patterns and analyze the relationship between modules and specific features. Candidate genes were screened by module membership (MM) > 0.8 and gene significance (GS) > 0.4, with both threshold of *P*-value < 0.05. Then these candidate genes were intersected between TCGA and TARGET data. Gene co-expression modules were identified using the *WGCNA* package.

### Identification of hub genes

Based on those intersected genes, AML patients’ overall survival prognosis model was constructed using TCGA data by univariate and multivariate cox regression methods. Univariate cox regression analysis for survival-related genes was determined by *P* < 0.05. Finally, genes included in the prognosis model were defined as hub genes. The analysis was based on *survival* and *survminer* packages.

### Relationship between hub genes and immune activity in TME

To investigate the correlation of hub genes and immune activity in microenvironment, the single-sample gene set enrichment analysis (*ssGSEA*) algorithm, which standardizes the gene expression value of an AML sample by rank and calculates the enrichment fraction of 29 immune cell types (23) using the empirical cumulative distribution function (24), was implemented to group AML samples into different immune activity sets. Patients were assigned into high- and low-immune activity groups using the *GSVA* package.

Furtherly, the ESTIMATE method (25), an algorithm designed to calculate scores for reflecting the infiltration levels of immune cells and stromal cells within the TME on the foundation of their specific genes expression level, was introduced to validate the accuracy of immune activity grouping based on the TCGA data. Then the relationship between hub genes expression level and immune activity was determined.

### Detection of hub genes at the single-cell level

Commonly, accurate positioning of gene expression in certain cells will contribute to a better understanding of their functional mechanism, and single cell RNA sequencing (scRNA) technology provides us a chance to realize the research.

Herein, the Tumor Immune Single-cell Hub (TISCH) (http://tisch.comp-genomics.org/home/), a database that integrated enormous scRNA data of various tumors, was used to obtain the accurate expression location of these genes. AML_GSE116256 (26) and PBMC_30K_10× datasets (a test data from 10× Genomics website) for AML and healthy control were obtained. Then, the immune subset of malignant cells in which genes are mainly expressed was explored to determine the precise mechanisms of action.

### Functional enrichment analysis of hub genes

To find the function of these hub genes, gene set enrichment analysis (GSEA) was performed based on their expression level to complete gene ontology (GO) and the Kyoto Encyclopedia of Genes and Genomes (KEGG) pathway enrichment analyses. According to the screening criteria suggested by MsigDB website (https://www.gsea-msigdb.org/gsea/msigdb), enrichment terms were included with *NOM p-value* < 0.05 and *FDR q-value* < 0.25. GSEA was performed by gsea software (version 3.0) and visualization was based on the *clusterProfiler* package.

### Pseudogene exploration

A pseudogene was included as a hub gene in this study. Pseudogenes are a special type of long non-coding RNAs (lncRNAs) that regulate different tumorigenic processes. To detect its function involved in competing for endogenous RNA (ceRNA) reaction in AML, we firstly retrieved its sequence information from the UCSC Genome Browser (http://genome.ucsc.edu/) and predicted its location in lncLocator (http://www.csbio.sjtu.edu.cn/bioinf/lncLocator/) and iLoc-LncRNA (http://lin-group.cn/server/iLoc-LncRNA/predictor.php) databases. Then its predicted mRNA sequence was put in miRDB (http://www.mirdb.org/) database to select its sponge-binding miRNAs.

LncRNAs are confirmed to be critical in influencing transcription factors involved in biological processes, therefore yielding or facilitating tumor development. We downloaded a gene set containing 318 tumor-related transcription factors (**Supplementary Table 1**) from the Cistrome Cancer (http://cistrome.org/CistromeCancer/) database, mapped them to TCGA expression data, and resulted in pseudogene-related transcription factors using the univariate Cox regression analysis method. Genes with a correlation coefficient > 0.5 and *P* < 0.05 were selected.

Additionally, the catRAPID omics database (http://service.tartaglialab.com/page/catrapid_group) was used to find potential genes interacting with the pseudogene. Moreover, pseudogene-related transcription factors and potentially related genes were intersected and put into the miRDB database to find potentially interacting miRNAs. Finally, pseudogene- and intersected transcription factors-related miRNAs were further intersected to determine those genes involved in the ceRNA mechanism.

### Validation of genes in peripheral blood by qRT-PCR

Based on these findings, we collected ethylen-ediaminetetracetic acid disodium (EDTA-Na_2_) anticoagulant peripheral whole blood of 24 AML patients and 19 healthy controls from the Children’s Hospital of Nanjing Medical University and The First Affiliated Hospital of Xi’an Jiaotong University from November 2020 to February 2021. All patients were initially diagnosed with AML; their blood was obtained before treatment. For mRNA and lncRNA validation, total RNA was extracted using the RNAprep Pure Hi-Blood Kit (TianGen Biotechnology, China) and reverse transcribed using the PrimeScript RT reagent kit (Takara, Japan). Primer sequences, which were synthesized by Generay Biotechnology (Shanghai, China), are listed in **Supplementary Table 2**. For microRNA validation, the specific primers of real-time reverse transcription PCR (qRT-PCR) from Bulge-loop™ miRNA qRT-PCR Primer Sets (one RT primer and a pair of qPCR primers for each set) for hsa-miR-6748-3p quantification were designed by RiboBio (Guangzhou, China).

Quantification of all genes was based on a real-time fluorescent quantitative PCR assay. The SYBR Green dye was purchased from Takara (Japan). For mRNA and lncRNA, GAPDH was used as an endogenous control gene; U6 was used as an endogenous control for miRNA. The relative quantification for gene expression was defined as 2^-ΔΔCT^ compared with control group. All PCR reactions were performed in triplicate.

### Dual-luciferase reporter gene assay and ceRNA mechanism validation

This study was performed in human embryonal kidney 293T cell line, CD4+ T cell and CD8+ T cell. CD4+ and CD8+ T cells were extracted from EDTA-Na_2_ anticoagulant peripheral whole blood (donated by Daheng Yang), using MojoSort™ Human CD4T/CD8T Cell Isolation Kit (BioLegend, USA). 293T cells and T cells were maintained in Dulbecco’s Modified Eagle’s medium (DMEM, Hyclone, USA) and 1640 medium (Hyclone, USA), respectively, with 10% fetal bovine serum (Gibco, USA), 100 U/ml penicillin and 100 U/ml streptomycin (Beyotime, China), and incubated at 5% CO_2_ at 37°C.

PmirGLO-CBX7 vector plasmids (mutant and wild types) were constructed by Generay Biotechnology (Nanjing, China); micrON™ miR-6748-3p mimic, micrOFF™ miR-6748-3p inhibitor and their negative controls were synthesized by RiboBio; overexpression plasmid and shRNA plasmid of IFITM3P6 were constructed by Qingke Biotech (Beijing, China), target sequences were presented in **Supplementary Table 2**. All experiments were performed in 6-well plates (Corning, USA) in triplicates. Synthetic nucleic acids were transfected by lipofectamine 2000 (Invitrogen, USA) to cells. Reporter gene assay was performed using the Dual-Luciferase^®^ Reporter Assay System Kit (Promega, USA) according to the manufacturer’s instructions, plasmids were quantified by 4μg and miR-6748-3p mimic was quantified by 100nM per well. Upregulation and downregulation of miR-6748-3p were quantified by 100nM mimic and 150nM inhibitor per well, respectively. Overexpression and down expression of IFITM3P6 plasmids were quantified by 4μg, respectively. CBX7 expression levels were detected by qRT-PCR with GAPDH as endogenous control in triplicates.

### RNA N^6^-methyladenosine (m6A) and DNA methylation analysis

RNA m6A and DNA methylation are important biological processes affecting expression of genes. Their abnormalities will lead to different consequences in tumor progression. Our study has included hub genes and their relevant genes, but aberrant expression mechanism is need to be elucidated. Methylation may be the main reason.

Herein, we downloaded DNA methylation data of 140 AML samples from the TCGA database using gdc-client tool (downloaded from https://gdc.cancer.gov/access-data/gdc-data-transfer-tool), and extracted methylation positions for each sample.

For RNA m6A methylation analysis, we compared the correlations of included genes with m6A-related genes by Pearson test. For DNA methylation analysis, correlations of gene expression with methylation and gene expression with methylation position were tested. All statistical significance was determined by Pearson coefficients > 0.3 or < -0.3, and *P* < 0.05.

### Statistical analysis

All statistical analyses were performed using R software (version 4.0.3) and RStudio (version 1.2.1335). The Wilcox signed-rank test was used for extracting differentially expressed genes (DEGs) with a |*log_2_Fold Change (FC)*| > 2 and adjusted *P-value* < 0.01. PCR detection was analyzed by unpaired t-test using GraphPad Prism 8 software (version 8.0.1), with a *P* < 0.05.

## Results

### Data preprocessing and identification of DEGs

The flow chart of the study is shown in the **Supplementary Figure 1**. Clinical information from the TCGA and TARGET datasets is shown in **Table 1**. There were 151 samples from 151 patients in TCGA and 358 samples from 296 patients in the TARGET cohort. After data batch correction by the normalize BetweenArrays function of the *limma* package, principal component analysis (PCA) showed that the tumor samples were significantly different from the control samples (**Supplementary Figures 2A, B**). Heatmap of the top 1000 genes with the highest standard deviation (SD) values indicated that there were significant differences between groups (**Supplementary Figures 2C, D**). The results suggested that the batch effect of the corrected expression data could be ignored. The number of DEGs in TCGA and TARGET was 11084 and 8436, respectively. WGCNA analysis was based on these DEGs.

**Table 1:** Clinical characteristics of patients in TCGA and TARGET datasets.

Characteristics	Databases			
	TCGA		TARGET	
Age (year)	> 50	94	>12	122
	≤50	57	<=12	174
Gender	male	83	Male	159
	female	68	Female	137
Leukocyte (×10^9^/L)	>30	63	>30	182
	≤30	88	≤30	114
Blast cell (%)	>30	84	>30	270
	≤30	67	≤30	17
	NA	0	NA	9
FAB category	M0	15	M0	8
	M1	35	M1	37
	M2	38	M2	73
	M3	15	M3	0
	M4	29	M4	72
	M5	15	M5	54
	M6	2	M6	4
	M7	1	M7	9
	unknown	1	unknown	39
Survival status	alive	54	alive	158
	dead	97	dead	138

FAB, French-American-British.

### CD4+ and CD8+ T cells infiltration levels are associated with OS of AML patients

**Figure 1:**
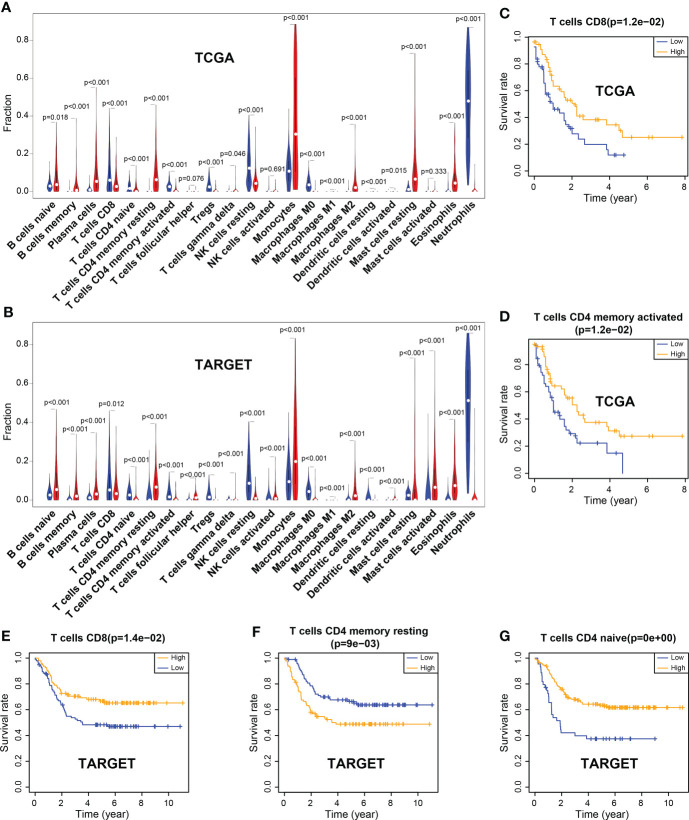
Differences of immune cell infiltration between the tumor and the normal group, and correlation between immune cells and the overall survival rate. **(A)** Violin plot of immune cell infiltration in TCGA. **(B)** Violin plot of immune cell infiltration in TARGET. **(C, D)** CD8 and activated memory CD4 T cells were associated with the overall survival (OS) rate, which was significantly higher in the high-level group than in the low-level group in TCGA. **(E–G)** CD8 T cells and naïve CD4 T cells were associated with the OS rate, which was markedly higher in the high-level group than in the low-level group in TARGET; however, resting memory CD4 T cells showed the opposite results.

CIBERSORT deconvolution algorithm showed that the infiltration of 22 types of immune cell was significantly different between the tumor group and the control group in TCGA (tumor group, n = 121; control group, n = 375) and TARGET (tumor group, n = 224; control group, n = 751) datasets. The results revealed that the infiltration level of resting memory CD4+ T cells was significantly increased in tumors. However, the infiltration levels of CD8+ T cells and naïve CD4+ T cells, activated memory CD4+ T cells were markedly reduced in tumors, both in TCGA and TARGET samples (**Figures 1A, B**; **Supplementary Figure 3**).

In the TCGA dataset, CD8+ T cells and activated memory CD4+ T cells were significantly associated with the patient’s survival. The OS rate of the high-level group was markedly higher than that of the low-level group (*P* = 0.012; *P* = 0.012). Similar results were observed for CD8+ T cells and naïve CD4+ T cells in the TARGET dataset (*P* = 0.014; *P*< 0.001). However, the infiltration of resting memory CD4+ T cells showed a contrary result (*P* = 0.009) (**Figures 1C–G**).

### Identification of hub genes

The WGCNA method was used to screen out genes related to infiltrating CD4+ and CD8+ T cells. By combining the CIBERSORT results and the differential gene expression matrices, outlier samples were deleted through sample clustering to reduce sample derived deviation. A soft threshold power of 4 was chosen for TCGA data to construct weighted genes co-expression networks, and 23 color modules were finally obtained by merging similar modules. Similarly, a soft threshold power of 8 was selected for TARGET data, and 21 color modules were finally obtained (**Supplementary Figure 4**).

**Figure 2:**
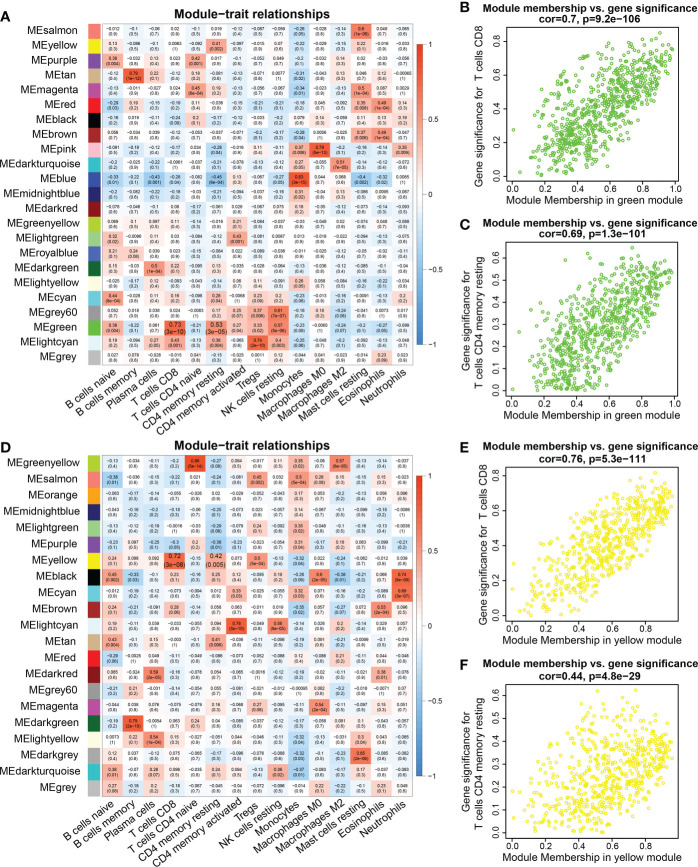
WGCNA analysis between genes and immune cell infiltration signatures. **(A, D)** Heatmap of the correlations between module eigengenes and immune cells infiltration. **(B)** Pearson correlation between gene significance (GS) in CD8 T cells and module membership (MM) in the green module in the TCGA database. **(C)** Pearson correlation between GS in memory CD4 T cells and MM in the green module in the TCGA database. **(E)** Correlation between GS in CD8 T cell and MM in the yellow module in the TARGET database. **(F)** Correlation between GS in resting memory CD4 T cells and MM in the yellow module in the TARGET database.

**Figure 3:**
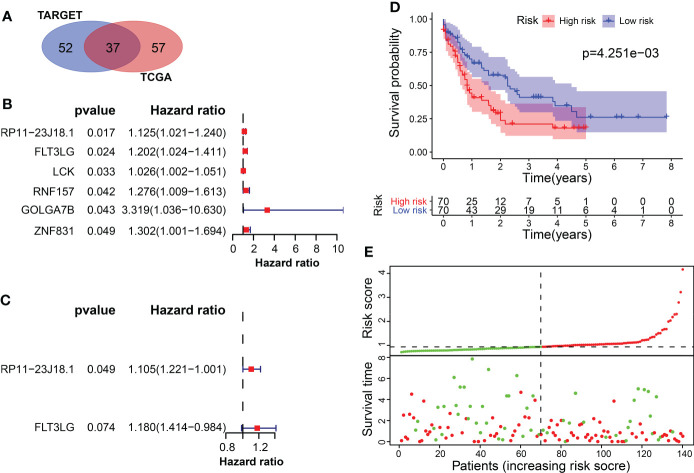
Screening of survival-related differentially expressed genes. **(A)** The intersection of candidate genes in TCGA and TARGET datasets. **(B)** Univariate cox regression analysis of 6 survival-related genes in TCGA. **(C)** Multivariate cox regression analysis included RP11-23J18.1 and FLT3LG as survival predicting gene models in TCGA. **(D, E)** Application of the survival predicting model in TCGA dataset.

Interestingly, the correlation analysis between the module genes and immune cells showed some similar patterns in the two datasets. In the TCGA dataset, the green module was significantly correlated with CD8+ T cells and resting memory CD4 T+ cells (R = 0.7, *P* = 9.2e-106; R = 0.69, *P* = 1.3e-101, respectively) (**Figures 2A–C**). In the TARGET dataset, the yellow module was significantly correlated with CD8+ T cells and resting memory CD4+ T cells (R = 0.76, *P* = 5.3e-111; R = 0.44, *P* = 4.8e-29, respectively) (**Figures 2D–F**). In addition, infiltration of CD4+ and CD8+ T cells was markedly associated with the OS rate of AML patients. Consequently, 94 genes were obtained from the TCGA dataset and 89 genes from the TARGET dataset, according to the screening criteria. The intersection of the two datasets included 37 candidate genes (**Figure 3A**).

The univariate Cox regression method screened 6 OS-related genes from these candidate genes using TCGA data, including RP11-23J18.1, FLT3LG, LCK, RNF157, GOLGA7B and ZNF831. All of the 6 genes were found to be downregulated in the tumor samples and were significantly associated with the OS rate of AML patients (**Figure 3B**, **Supplementary Figure 5**). Multivariate cox regression analysis was used to build a survival prediction model, which finally included RP11-23J18.1 and FLT3LG (**Figure 3C**). The Kaplan-Meier curve showed that the OS rate in the lower-risk group was significantly higher than that in the higher-risk group (*P* = 4.251e-03), suggesting that the patients’ overall survival time decreases with an increase of the risk score (**Figure 3D, E**).

### RP11-23J18.1 and FLT3LG are positively correlated with immune activity

**Figure 4:**
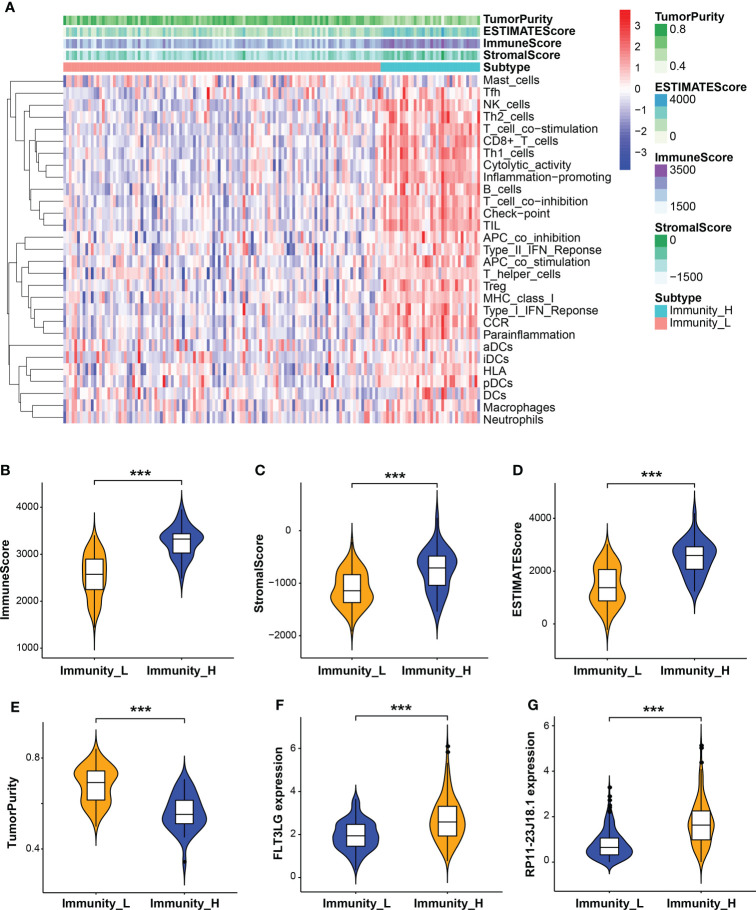
Evaluation of tumor microenvironment (TME). **(A)** Heatmap of immune cell, stromal cell and ESTIMATE scores and tumor purity in high and low immune activity groups. **(B–E)** Relationship between immune cell, stromal cell and ESTIMATE scores and tumor purity with immune activity in the TME. **(F, G)** Relationship between FLT3LG and RP11-23J18.1 expression and immune activity. ***p < 0.001.

Next, the relationship between RP11-23J18.1 and FLT3LG and immune activity was explored. Firstly, we utilized the ESTIMATE algorithm to evaluate the TCGA expression matrix, obtained the immune cell score, stromal cell score, ESTIMATE score (the total score) and tumor purity score in the TME (**Figure 4A**), finding that immune cell score, stromal cell score and ESTIMATE score were positively correlated with immune activity (**Figures 4B–D**), while tumor purity was inversely correlated with immune activity (**Figure 4E**). That validated high- and low-immune activity grouping by ssGSEA method was accurate.

**Figure 5:**
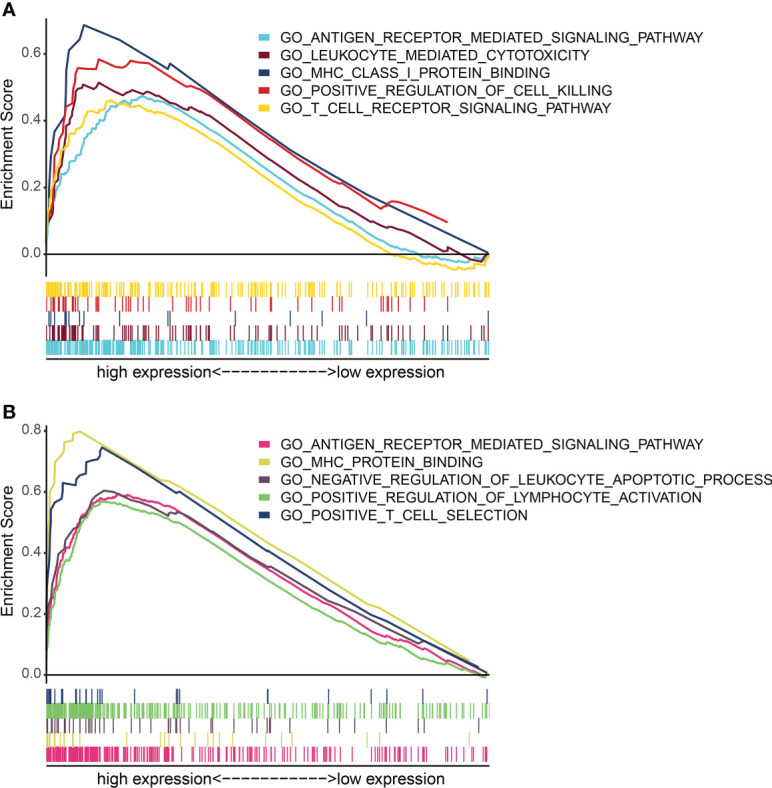
GO term enrichment analysis of FLT3LG and RP11_23J18.1 by GSEA method. **(A)** GO analysis of FLT3LG. **(B)** GO analysis of RP11_23J18.1.

Further analysis showed that the expression levels of FLT3LG and RP11-23J18.1 genes were significantly higher in the high immunocompetence group compared with the low immunocompetence group in the TCGA (*P* < 0.001; *P* < 0.001; **Figures 4F, G**). These results suggest that FLT3LG and RP11-23J18.1 may play an important role in the activation process of CD4+ and CD8+ T cells. Moreover, GSEA analysis showed that FLT3LG and RP11-23J18.1 were mainly enriched in antigen receptor-mediated signaling transduction, cell killing, T cell migration and differentiation, interleukin regulation, etc. (**Figure 5**). These biological processes are closely related to T cells, which are involved in killing tumor cells.

### FLT3LG-FLT3 axis involved in influencing dendritic cell activation

**Figure 6:**
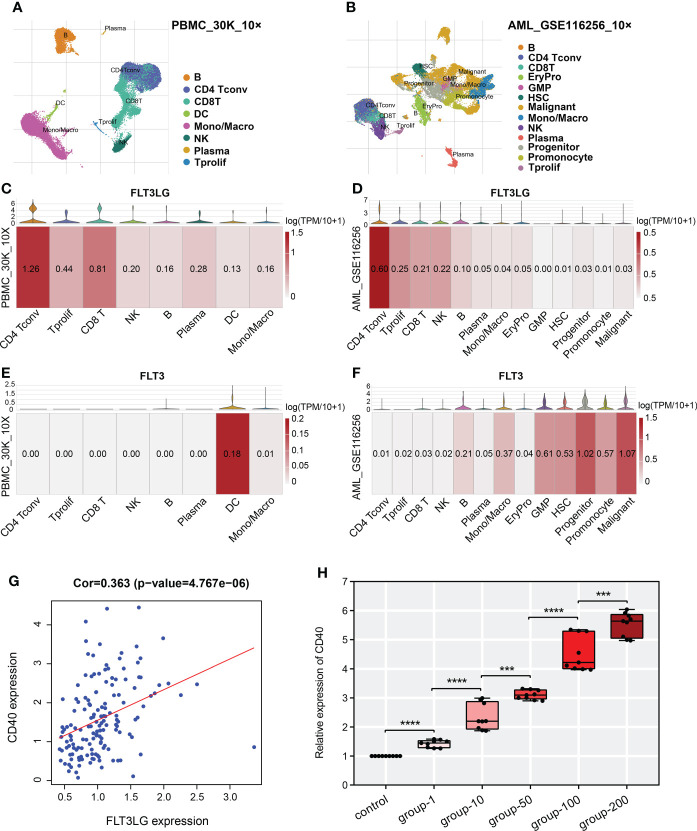
Single-cell transcriptome analysis revealed detailed gene location in various cell types. **(A, B)** UMAP cluster after cluster analysis of peripheral blood mononuclear cells (PBMC) and acute myelocytic leukemia (AML). **(C, E)** Violin plot and heatmap of FLT3LG and FLT3 expression in the PBMC_30K_10× dataset. **(D, F)** The transcription level of FLT3LG and FLT3 in cell types of the AML_GSE116256 dataset. **(G)** FLT3LG expression was positively correlated with CD40 expression in TCGA data. **(H)** CD40 expression was significantly elevated when Flt3lg cytokine activation in dendritic cells. ***p < 0.001; ****p < 0.0001.

In TISCH database, we retrieved FLT3LG is mainly expressed in CD4+ and CD8+ T cells in healthy control (PBMC_30K_10× dataset, **Figures 6A, C**). Its receptor, FLT3, is mainly expressed in dendritic cells (DCs) (**Figure 6E**). Pearson test determined FLT3LG expression was positively correlated with CD40 expression, which is the marker of dendritic cell activation (**Figure 6G**). In the AML_GSE116256 dataset, FLT3LG is mainly expressed in CD4+ T cells, but compared with the PBMC_30K_10× dataset, the expression level of FLT3LG is remarkably reduced (**Figures 6B, D**), and FLT3 is mainly expressed in leukemia cells and precursor cells (**Figure 6F**). This reminder us FLT3LG (ligand) may activate DCs by acting on FLT3 (receptor) under physiological conditions.

To prove it, we purchased primary dendritic cells (cat NO.: CP-R162) that were extracted from the bone marrow of rats by flow cytometry from Procell Life Science & Technology Corporation (Wuhan, China), cultured them in customized media (provided by the company). Added 1μg/ml, 10μg/ml, 50μg/ml, 100μg/ml and 200μg/ml of recombinant rat Flt3lg cytokine (PEPROTECH, USA) per well (6-well plate) to stimulate the activation of dendritic cells and performed qRT-PCR, found that expression of CD40 was higher than the negative control, and its expression presented positive dose-dependent (**Figure 6H**).

### IFITM3P6 influences CBX7 expression by sponge binding miR-6748-3p

The pseudogene, RP11-23J18.1 (ensemble name ENSG00000258352), was put into the UCSC Genome Browser (http://genome.ucsc.edu/) and GeneCards (https://www.genecards.org/) databases. It was confirmed that it mostly matched with IFITM3P6 (IFITM3P pseudogene 6). The annotation for its gene version (ENSG00000258352.1_8) and transcript version (ENST00000553227.1_1) was validated level. Therefore, the predicted RNA sequences of IFITM3P6 provided by the UCSC database were obtained (**Supplementary Table 3**). In the NCBI database, the gene ID of IFITM3P6 was confirmed to be 643058 (*Homo sapiens*). Transcriptome sequencing analysis showed that IFITM3P6 had the highest mean expression in blood compared with other tissues and organs in its physiologic context in the GTEx (https://www.gtexportal.org/home/gene/ENSG00000258352 ) database (**Supplementary Figure 6**). In subsequent studies, IFITM3P6 will be used as the pseudogene instead of RP11-23J18.1.

In the lncLocator database, the predicted subcellular locations of IFITM3P6 were mainly in cytoplasm and cytosol with a score of 0.449968 and the nucleus with a score of 0.416618. In the iLoc-LncRNA database, the subcellular location was cytoplasm and cytosol, with a probability score of 0.956631. Based on these results, we hypothesized that IFITM3P6 is a lncRNA that exerts function in the cytoplasm. A total of 579 IFITM3P6-related genes were retrieved from the catRAPID omics database (**Supplementary Table 4**), which are potentially binding to IFITM3P6 directly or indirectly.

**Figure 7:**
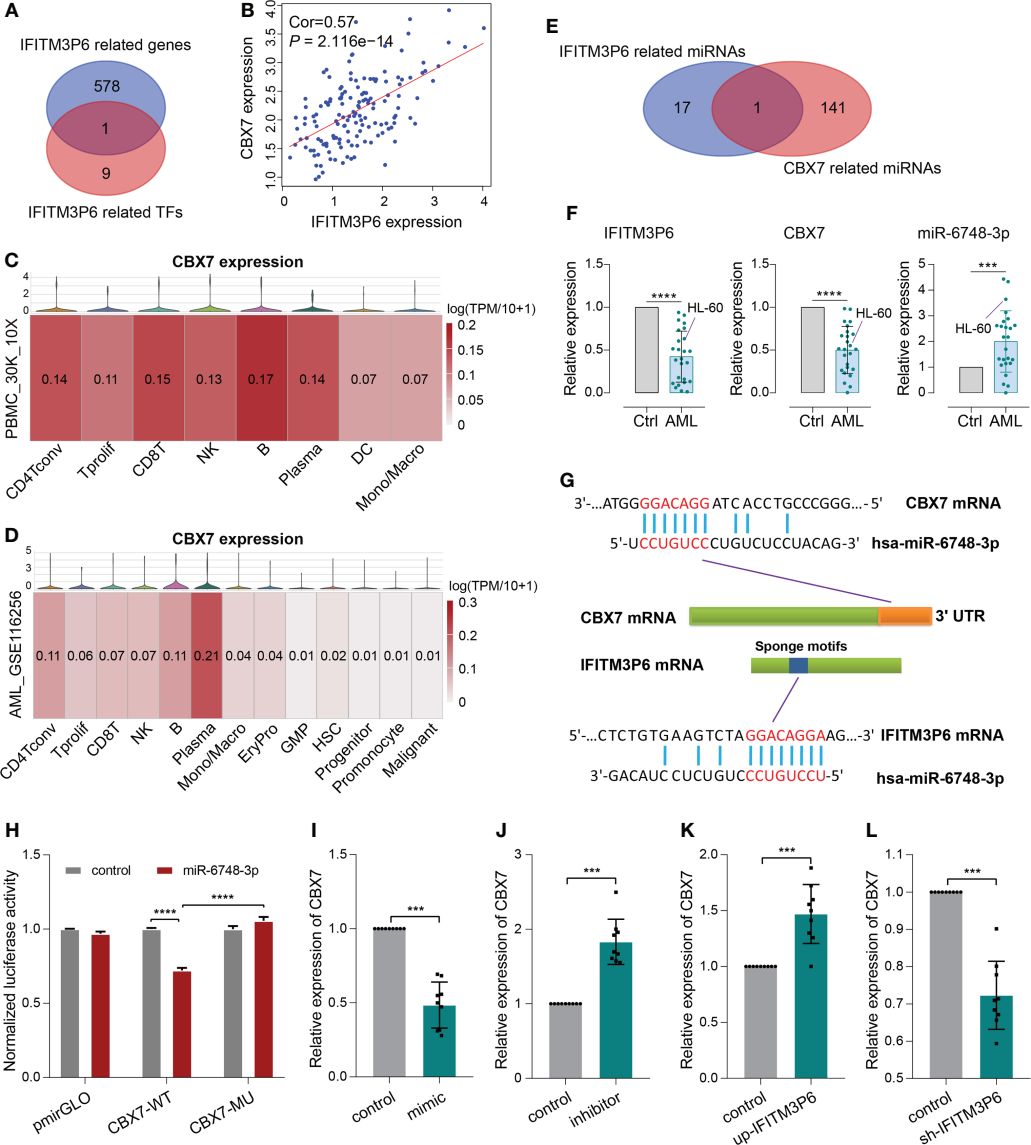
ceRNA mechanism detection of IFITM3P6 in T cell activation. **(A, B)** CBX7 was determined as a hub transcription factor that is computationally correlated with IFITM3P6. **(C, D)** Location of CBX7 in cell subtypes in healthy and AML single-cell transcriptome sequencing datasets. **(E)** Venn diagram of IFITM3P6- and CBX7-related miRNAs. **(F)** Relative expression of IFITM3P6, CBX7 and miR-6748-3p compared between the AML group and healthy control group. **(G)** Potential competing endogenous RNA (ceRNA) mechanism of IFITM3P6 and CBX7. **(H)** Dual-luciferase reporter assay revealed that miR-6748-3p could bind to the 3’ UTR of CBX7. **(I, J)** MiR-6748-3p expression was negatively correlated with CBX7 expression. **(K, L)** IFITM3P6 expression was positively correlated with CBX7 expression. ***p < 0.001; ****p < 0.0001.

Next, 10 transcription factors were identified, which were significantly correlated with IFITM3P6. Then, 579 IFITM3P6-related genes were intersected with the 10 IFITM3P6-related transcription factors, including CBX7 as a core gene (**Figure 7A**).

In TCGA data, the expression of IFITM3P6 was positively correlated with CBX7 expression level by Pearson test (R = 0.57, *P* = 2.116e-14) (**Figure 7B**). scRNA analysis showed that CBX7 was mainly expressed in T cells, B cells, NK cells and plasma cells in a healthy control, while it presented a striking decrease in the AML data (**Figures 7C, D**).

Furthermore, 18 IFITM3P6-related miRNAs and 142 CBX7-related miRNAs were predicted in the miRDB database. miR-6748-3p was finally intersected from them (**Figure 7E**; **Supplementary Table 5**). Compared to healthy controls, IFITM3P6 and CBX7 were significantly decreased in AML blood and HL-60 cell (t = 9.665, *P* < 0.0001; t = 9.043, *P* < 0.0001, respectively). However, miR-6748-3p was significantly increased in AML blood samples (t = 4.197, *P* < 0.001) (**Figure 7F**).

The binding sites of miR-6748-3p to CBX7 and IFITM3P6 were predicted in the miRDB database (**Figure 7G**). Dual-luciferase reporter assay in 293T cells confirmed that CBX7 wild type and miR-6748-3p mimic co-transfected group significantly decreased luciferase activity compared with CBX7 wild type and miR-6748-3p negative control co-transfected group (*P* < 0.0001). The result was comparable with CBX7 mutant type and miR-6748-3p mimic co-transfected group (*P* < 0.0001) (**Figure 7H**), while the luciferase activity of the co-transfected group with miR-6748-3p mimic and CBX7 mutant type had no significance compared to control group, suggesting that miR-6748-3p could bind to the 3’ UTR of CBX7, thereby interfering with its expression. Similar results were validated in CD4+ and CD8+ T cells (**Supplementary Figure 7**).

Compared with negative controls, mRNA of CBX7 presented significantly lower expression in miR-6748-3p mimic group, while presented higher expression in miR-6748-3p inhibitor group (**Figures 7I, J**). Furtherly, qRT-PCR revealed a higher expression of CBX7 compared with negative control when IFITM3P6 was upregulated, while its downregulation led contrary result (**Figures 7K, L**).

### RNA m6A and DNA methylation regulate gene expression

**Figure 8:**
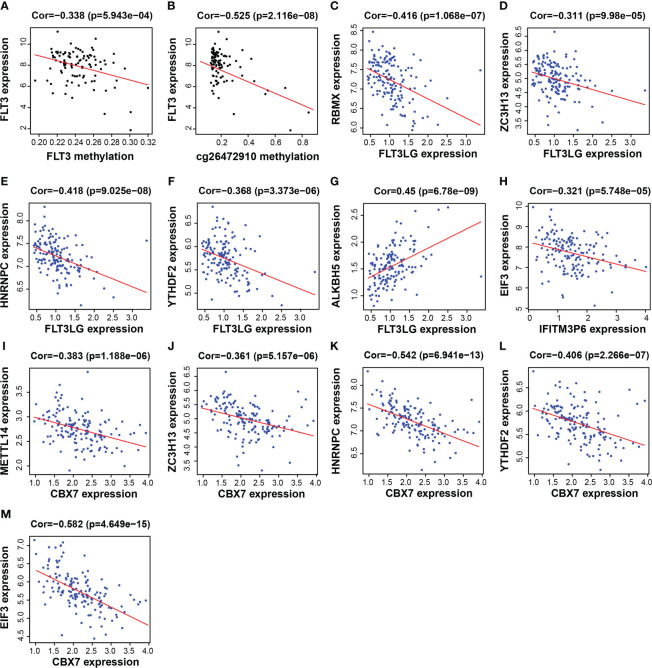
Functional mechanism analysis for FLT3LG, IFITM3P6 and CBX7. **(A, B)** Relationship between FLT3 DNA methylation and FLT3 expression and the main methylation site. **(C–G)** Relationship between FLT3LG expression and N^6^-methyladenosine (m6A) methylation-related gene expression. **(H)** Relationship between IFITM3P6 expression and m6A methylation-related gene expression. **(I–M)** Relationship between CBX7 expression and m6A methylation-related gene expression.

FLT3LG, IFITM3P6 and CBX7 were downregulated in AML samples, while FLT3 was upregulated. The mechanism underlying the cause of aberrant gene expression is worthy of exploring. We found that FLT3 expression is negatively correlated with its DNA methylation level (R = -0.338, *P* = 5.943e-04) (**Figure 8A**), in which the main methylation site is cg26472910 (**Figure 8B**). However, FLT3LG, IFITM3P6 and CBX7 expression presented non-significant correlations with their DNA methylation level.

Furthermore, the relationship between the expression of these genes and m6A-related gene expression was assessed, found that FLT3LG was negatively correlated with m6A writer and m6A reader genes, including RBMX (writer, R = -0.416, *P* = 1.068e-07, **Figure 8C**), ZC3H13 (writer, R = -0.311, *P* = 9.98e-05, **Figure 8D**), HNRNPC (reader, R = -0.418, *P* = 9.025e-08, **Figure 8E**) and YTHDF2 (reader, R = -0.368, *P* = 3.373e-06, **Figure 8F**), but positively correlated with m6A eraser gene, ALKBH5 (R = 0.45, *P* = 6.78e-09, **Figure 8G**). IFITM3P6 was negatively correlated with m6A reader gene, EIF3 (R = -0.321, *P* = 5.748e-05, **Figure 8H**). CBX7 was negatively correlated with m6A writer and m6A reader genes, including METTL14 (writer, R = -0.383, *P* = 1.188e-06, **Figure 8I**), ZC3H13 (writer, R = -0.361, *P* = 5.157e-06, **Figure 8J**), HNRNPC (reader, R = -0.542, *P* = 6.941e-13, **Figure 8K**), YTHDF2 (reader, R = -0.406, *P* = 2.266e-07, **Figure 8L**) and EIF3 (reader, R = -0.582, *P* = 4.649e-15, **Figure 8M**).

### FLT3LG, IFITM3P6 and CBX7 may exist coupling effects

**Figure 9:**
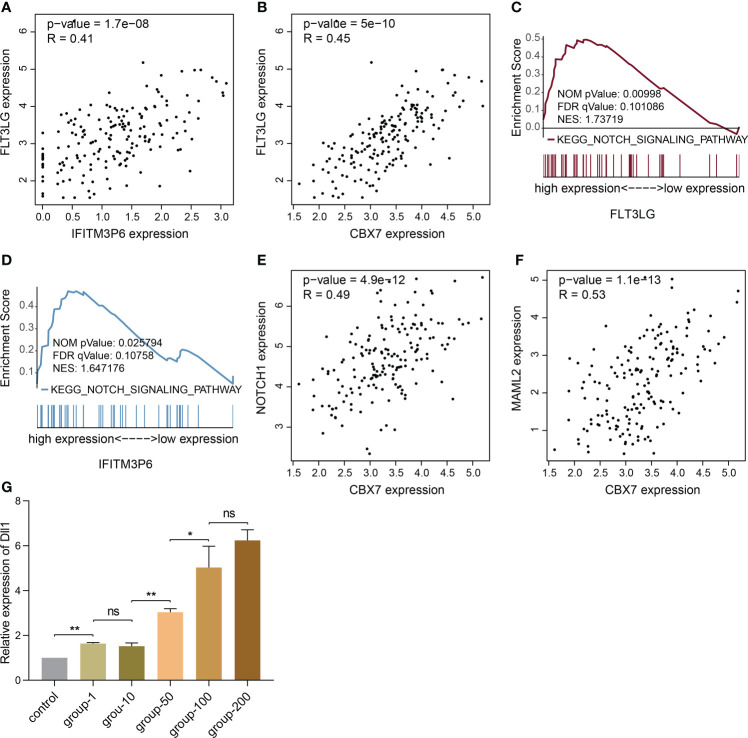
The potential relationship of FLT3LG and IFITM3P6 in activating T cells. **(A)** FLT3LG is significantly correlated with IFITM3P6 in TCGA data. **(B)** FLT3LG is positively correlated with CBX7. **(C, D)** FLT3LG and IFITM3P6 are enriched in NOTCH signaling pathway. **(E, F)** CBX7 is positively correlated with NOTCH1 and MAML2. **(G)** Dll1 is increased in rat bone marrow-derived dendritic cells along with elevated concentration (μg/ml) of Flt3lg stimulation. *p < 0.05; **p < 0.01; ns, no significance.

Now that FLT3-FLT3LG and IFITM3P6-miR-6748-3p-CBX7 axes were determined in activating T cells, we were curious about whether they exist coupling effects. Interestingly, we found FLT3LG was positively correlated with IFITM3P6 (R = 0.41, *P* = 1.7e-08, **Figure 9A**) and CBX7 (R = 0.45, *P* = 5e-10, **Figure 9B**) in TCGA data. It’s suggested that CBX7 may be the key node connecting FLT3LG and IFITM3P6.

Furtherly, KEGG enrichment analysis by GSEA method showed high expression of FLT3LG and IFITM3P6 were both enriched in NOTCH signaling pathway (**Figures 9C, D**). Meanwhile, CBX7 was determined to be positively correlated with NOTCH1 (R = 0.49, *P* = 4.9e-12, **Figure 9E**) and MAML2 (R = 0.53, *P* = 1.1e-13, **Figure 9F**).

NOTCH pathway has been well documented in activating CD4+ and CD8+ T cells. CBX7 was reported to involve in FasL suppression, the latter is an important factor suppressing T cell activation. This reminded us that NOTCH1, MAML2 and CBX7 in nucleus may form a suppressive complex to suppress FasL expression. In PPA-Pred2 database (https://www.iitm.ac.in/bioinfo/PPA_Pred/), a protein-protein affinity predictor, we retrieved their potential binding affinity by putting their protein sequences, and predicted value of Delta G (ΔG, binding free energy) between CBX7 and NOTCH1 is -12.58 kcal/mol, between CBX7 and MAML2 is -16.91 kcal/mol, between NOTCH1 and MAML2 is -30.07 kcal/mol. These provide evidence that CBX7, NOTCH1 and MAML2 exist binding potential to reduce FasL expression.

Ligand-receptor reaction is the indispensable condition for signaling pathway activation. Herein, we found Dll1 (delta like 1), the ligand of NOTCH receptor, in rat bone marrow-derived dendritic cells was significantly increased in Flt3lg-stimulating model, with a trend of dose-dependent (**Figure 9G**), revealing that activated dendritic cells, which is stimulated by FLT3LG-FLT3 axis, will further activate NOTCH pathway in T cells.

## Discussion

AML is a biologically and clinically heterogeneous disease, despite advances in supportive care and prognostic risk stratification that have been optimized for established treatments, overall long-term survival remains a challenge (3). Leukemia blasts exhibit impressive immunoediting capabilities under the selective immune pressure. The presence of T cells at the tumor site is the prerequisite for immune recognition and elimination of AML cells, also for any therapy leveraging on this condition. In a mouse model of AML, deletional CD4+ and CD8+ T cell tolerance induction is attributed to leukemia antigen presentation by immature antigen-presenting cells (DCs) or splenic CD8α+ dendritic cells (27, 28). Moreover, a recent study demonstrated loss of plasmacytoid DC differentiation was associated with persistence of the residual disease after AML treatment and unfavorable outcomes (29).

Our present research and those previously reported studies have confirmed that FMS-related tyrosine kinase 3 (FLT3) is mainly physiologically expressed in DCs (30), but pathologically expressed in malignant and progenitor cells. FLT3 ligand (FLT3LG) is predominantly produced by lymphocytes, especially T cells (31). Admittedly, FLT3LG has been shown to bind to FLT3 (receptor) on DCs to stimulate their differentiation and expansion, facilitating tumor antigen cross-presentation and anticancer immune responses (32). However, in a malignant context, FLT3 mutations induce receptor dimerization, resulting in constitutive activation of PI3K-AKT, RAS-MEK-MAPK and STAT-5 signaling pathways (33).

We found that low DNA methylation occurred in AML, induced upregulation of FLT3, that’s a reason for eliciting pro-tumor signaling pathway activation. Targeting the methylation site (cg26472910) may lead to the elimination of FLT3 upregulation. FLT3LG expression was positively correlated with the N^6^-methyladenosine (m6A) eraser gene (ALKBH5) but negatively correlated with the writer (RBMX, ZC3H13) and reader (HNRNPC, YTHDF2) genes, and induced its low expression. Considering FLT3LG is positively correlated with immune activity in the TME, this might be one of the reasons for the attenuated power of DCs in tumor antigen presentation. Regarding DCs function, it’s been well documented as the strongest presenting cell in activating T cells. Hence, FLT3LG-FLT3 axis is a key way to promote DC-based cross-priming of antileukemia T cells.

IFITM3P6 was identified as a special type of lncRNA that regulates AML processes and positively correlated with immune activation in this study. It was predicted to be mainly in the cytoplasm and hypothesized that it may exert functions *via* the ceRNA mechanism. Based on these findings and analysis of multiple databases, we found that the IFITM3P6-miR-6748-3p-CBX7 axis may play an important role in regulating the activity of T cells.

Chromobox homolog 7 (CBX7) is a polycomb protein involved in the formation of polycomb repressive complex 1. Low expression of the CBX7 gene is associated with poor prognosis in most cancers (34). It has been reported that CBX7 represses FasL expression in CD4+ T cells, consequently preventing CD4+ T cell apoptosis (35). Here, we also found that CBX7 expression was negatively correlated with caspase 3 expression (data not presented), suggesting that the IFITM3P6-miR-6748-3p-CBX7 axis modulates T cells activity and apoptosis.

M6A is methylation that occurs in the N^6^-position of adenosine, which is the most prevalent internal modification in eukaryotic mRNA. Accumulating evidence suggests that m6A modulates gene expression (36). We found that EIF3 (m6A reader) was reported to be upregulated in AML (37) and was negatively correlated with IFITM3P6, suggesting that m6A modification could reduce the expression of IFITM3P6. m6A writer and reader genes, including METTL14 (writer), ZC3H13 (writer), HNRNPC (reader) and EIF3 (reader) were also negatively correlated with CBX7 expression. In addition, they are upregulated in AML (38, 39). METTL14 and ZC3H13 are accessory subunits that form a stable complex with METTL3 and play key roles in substrate recognition (40–42). HNRNPC is mainly involved in mediating mRNA splicing (43). Consequently, m6A modification in combination with IFITM3P6-miR-6748-3p-CBX7 regulatory axis downregulates CBX7 in AML, thereby prompting T cells apoptosis.

In a summary, the downregulation of FLT3LG induced by m6A modification and the ectopic expression of FLT3 in malignant cells and progenitor cells ultimately decrease the ability of DCs to present antigen to T cells. What’s more, m6A modification induces the downregulation of IFITM3P6 to regulate the expression of CBX7 through the ceRNA mechanism, thereby inducing T cell apoptosis in AML. Additionally, we found that FLT3LG and IFITM3P6 were both enriched in NOTCH signaling pathway. Evidence showed they may a exert synergy effect by promoting the formation of NOTCH1-MAML2-CBX7 suppressive transcription system, combining with the canonical pathway molecule like RBP-Jк, to block FasL expression.

The role of IFITM3P6-miR-6748-3p-CBX7 and FLT3LG-FLT3 axes in AML was revealed for the first time, but the experimental verification was not solid. Whether these key genes could exert function on AML cells directly, such as the landscape of exosome of IFITM3P6 and/or miR-6748-3p secreted by T cells to interact with tumor cells, these questions remain to be explored. What’s more, the FLT3 gene located near the membrane region mutation has been found in about 30% of patients with AML. It is an internal tandem duplication (ITD) mutation composed of exon 11, intron 11 and exon 12, which is called the FLT3/ITD gene mutation. The impact of ITD on FLT3 was not evaluated in the current study. FLT3-TKD (tyrosine kinase domain) is another form of mutation. These two mutations are associated with recurrence and drug resistance.

**Figure 10:**
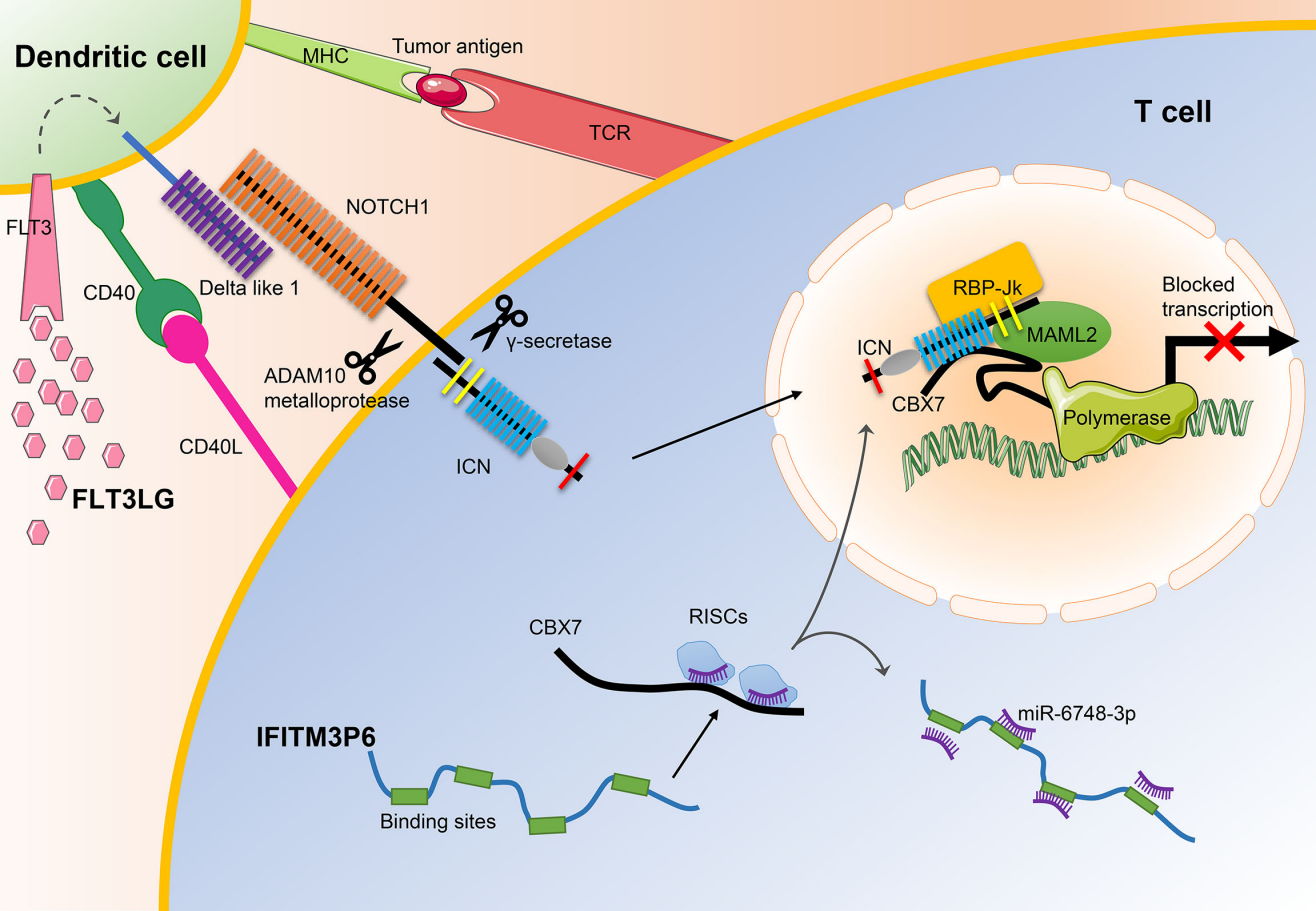
The main mechanisms of FLT3LG and IFITM3P6 in T cell activation. On the one hand, increased DLL1 by stimulation of FLT3LG to FLT3 in dendritic cells, transduct signals to NOTCH1 receptor expressed by T cells. Ligand/receptor binding triggers sequential proteolytic cleavage of NOTCH receptor, first by the ADAM10 metalloprotease and by the γ-secretase complex. These cleavages release intracellular NOTCH (ICN) into the nucleus. On the other hand, IFITM3P6 as a kind of ceRNA inhibit targeted degradation of CBX7 by miR-6748-3p. They finally promote the formation of NOTCH1-MAML2-CBX7 complex system, and suppress expression of FasL.

In general, our results demonstrate that IFITM3P6 and FLT3LG might serve as prognostic markers in AML and may be used as potential therapeutic targets for the treatment of leukemia in the future (**Figure 10**).

## Data availability statement

The original contributions presented in the study are included in the article/**Supplementary Material**. Further inquiries can be directed to the corresponding author.

## Ethics statement

The studies involving human participants were reviewed and approved by Ethics committee of Children’s Hospital of Nanjing Medical University. Written informed consent to participate in this study was provided by the participants’ legal guardian/next of kin.

## Author contributions

HC: Conceptualization, methodology, software, writing-original draft, supervision, writing-review & editing. MW: Methodology, validation, investigation, writing-original draft, funding acquisition. HX: Writing-review & editing, visualization, funding acquisition. SD: Methodology, investigation, validation. GL: Formal analysis. GZ: Data curation, software, writing-review & editing, funding acquisition. YZ: Methodology, software. XH: Data curation. DY: Conceptualization, methodology, software, data curation, supervision, writing-original draft, writing-review & editing, project administration, funding acquisition. All authors contributed to the article and approved the submitted version.

## Funding

This study was supported by Academic Technology Program of Nanjing Medical University (No. NMUB2020096) and Nanjing Medical Key Science and Technology Development Project Fund (No. YKK19105), The Recruitment Program of Overseas High-Level Young Talents, “Innovative and Entrepreneurial Team” (No. (2018)2015), Science and Technology Grant of Jiangsu Province (BE2019758), Beijing Xisike Clinical Oncology Research Foundation, Shaanxi Province Hundred Talents and ‘Young Top Talent’ of Xi’an Jiaotong University.

## Acknowledgments

We thank Dr. Jianming Zeng (University of Macau), and all the members of his bioinformatics team, biotrainee, for generously sharing their experience and codes.

## Conflict of interest

The authors declare that the research was conducted in the absence of any commercial or financial relationships that could be construed as a potential conflict of interest.

## Publisher’s note

All claims expressed in this article are solely those of the authors and do not necessarily represent those of their affiliated organizations, or those of the publisher, the editors and the reviewers. Any product that may be evaluated in this article, or claim that may be made by its manufacturer, is not guaranteed or endorsed by the publisher.
