# Supplementary figures and images for: FLT3LG and IFITM3P6 consolidate T cell activity in the bone marrow microenvironment and are prognostic factors in acute myelocytic leukemia
Source: Front Immunol. 2022 Aug 23;13:980911. doi: 10.3389/fimmu.2022.980911 (PMC9445253; doi:10.3389/fimmu.2022.980911)

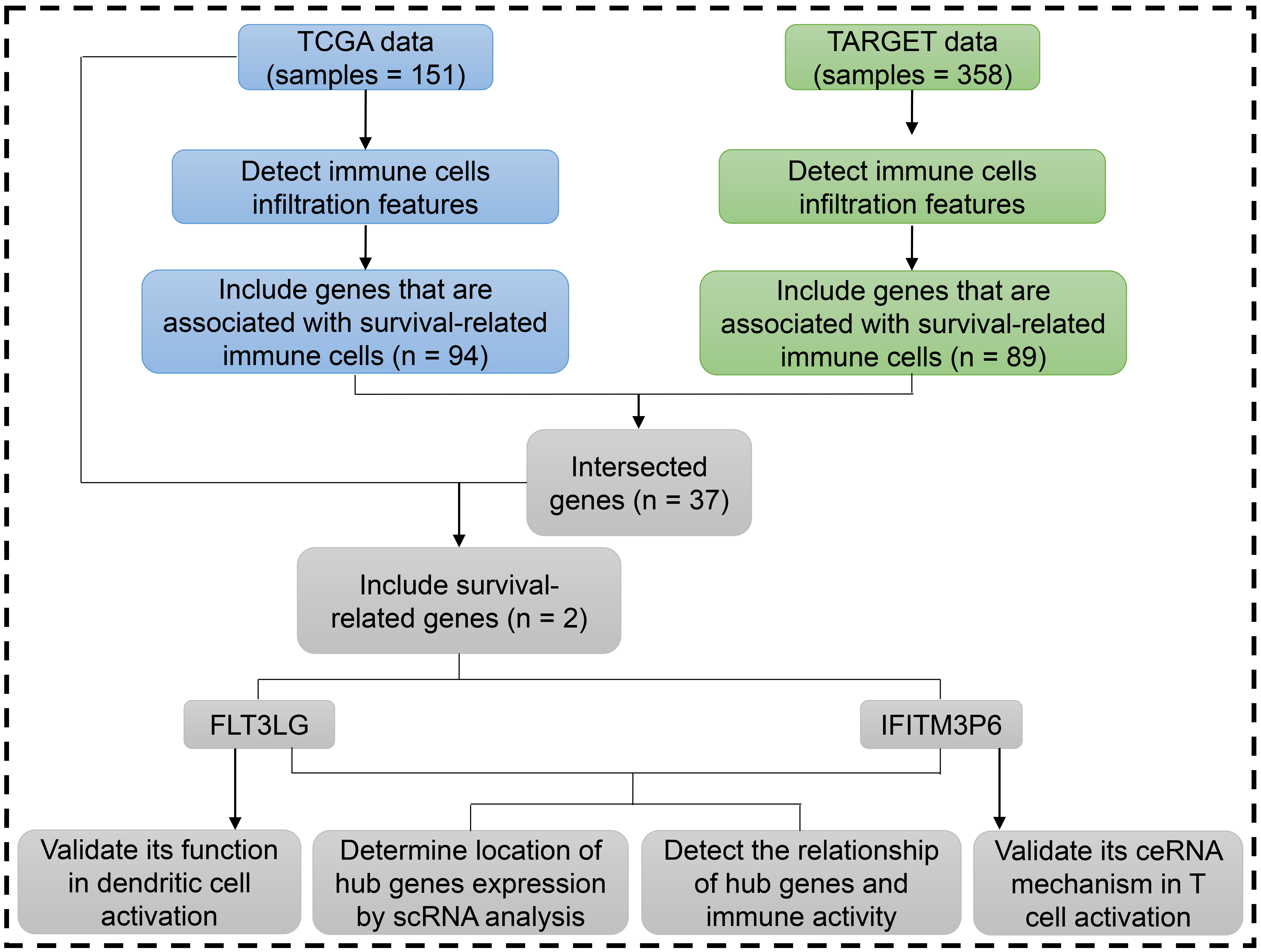

Supplement: Supplementary file 1 [file Image_1.tif]

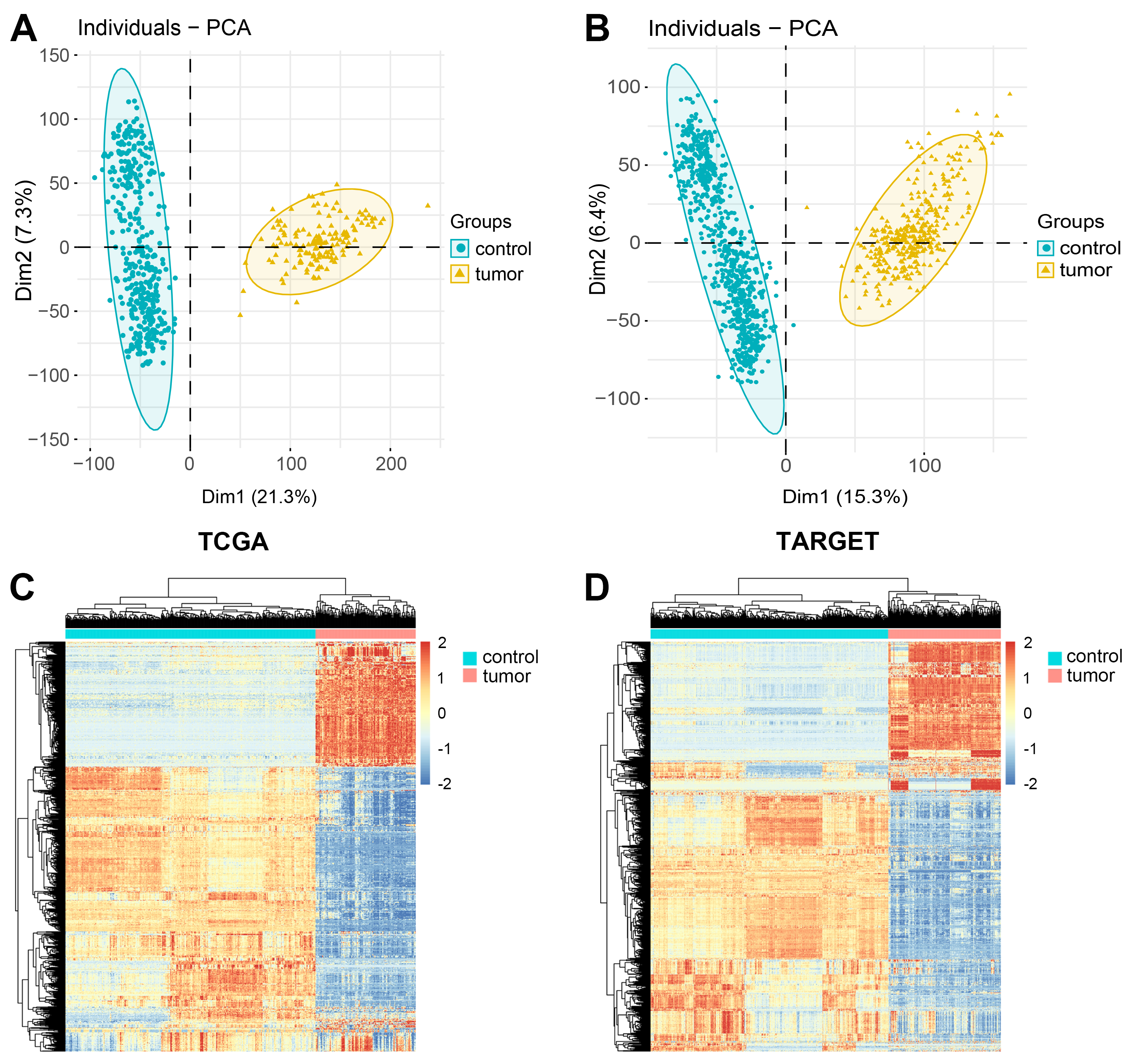

Supplement: Supplementary file 2 [file Image_2.tif]

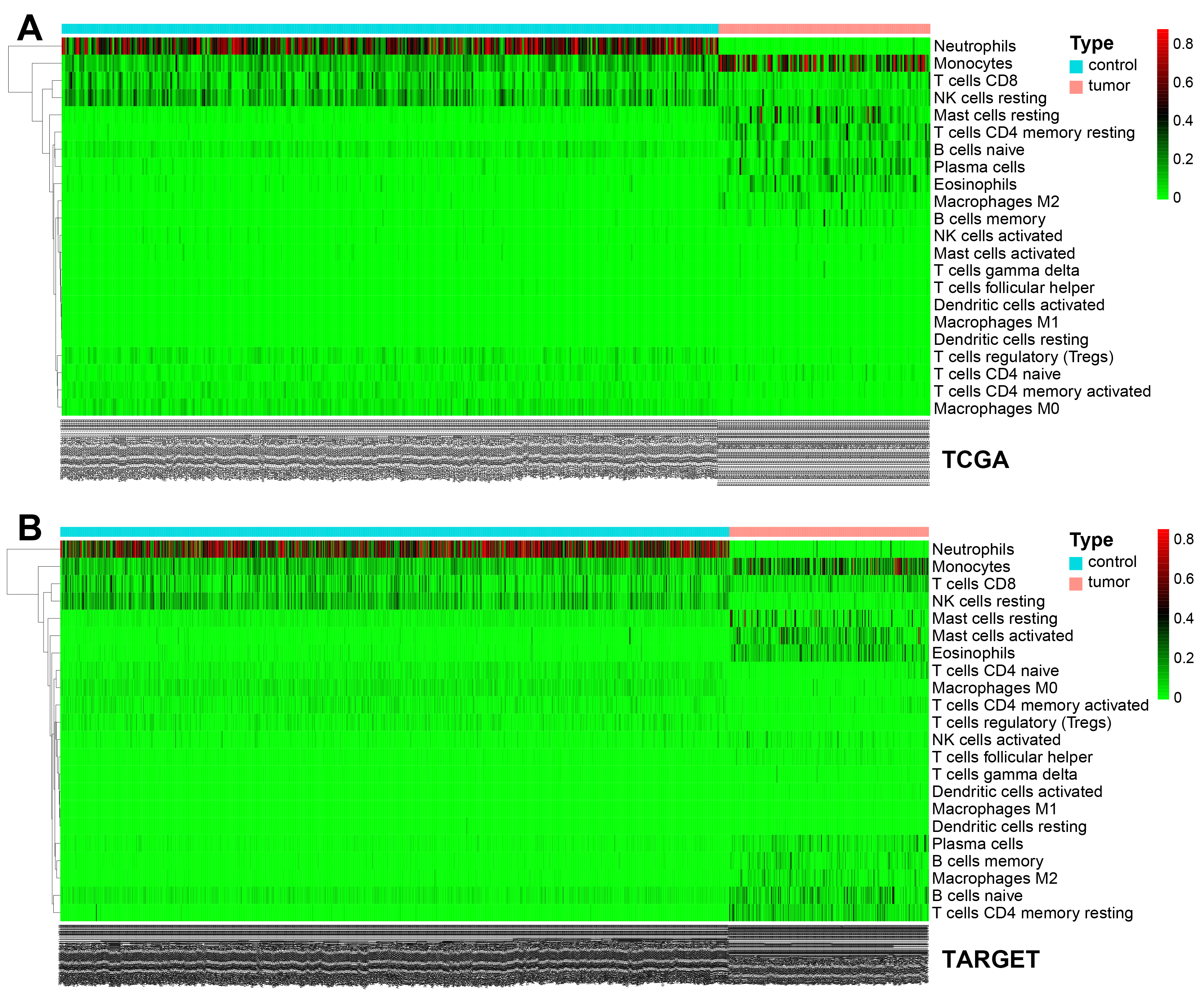

Supplement: Supplementary file 3 [file Image_3.tif]

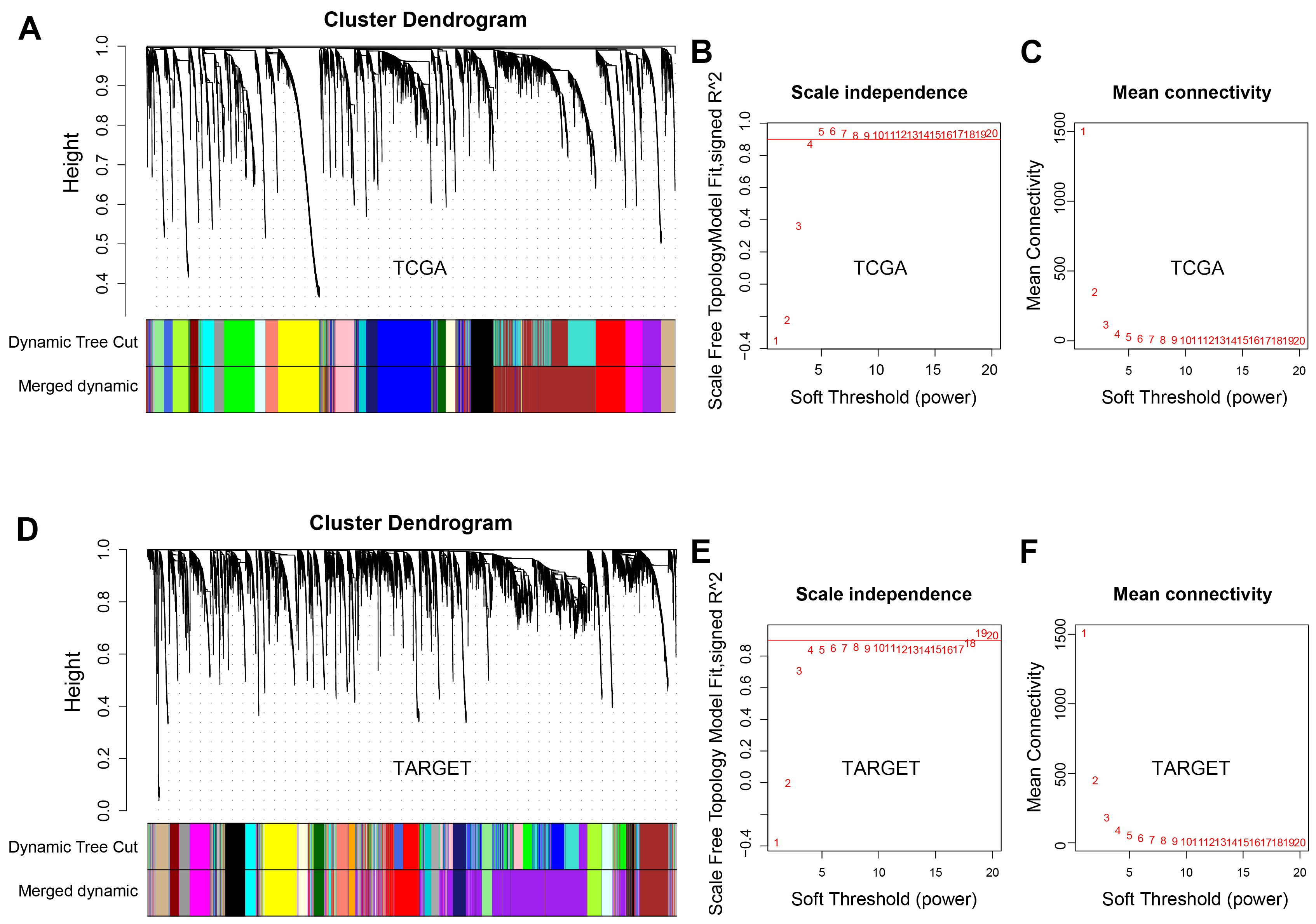

Supplement: Supplementary file 4 [file Image_4.tif]

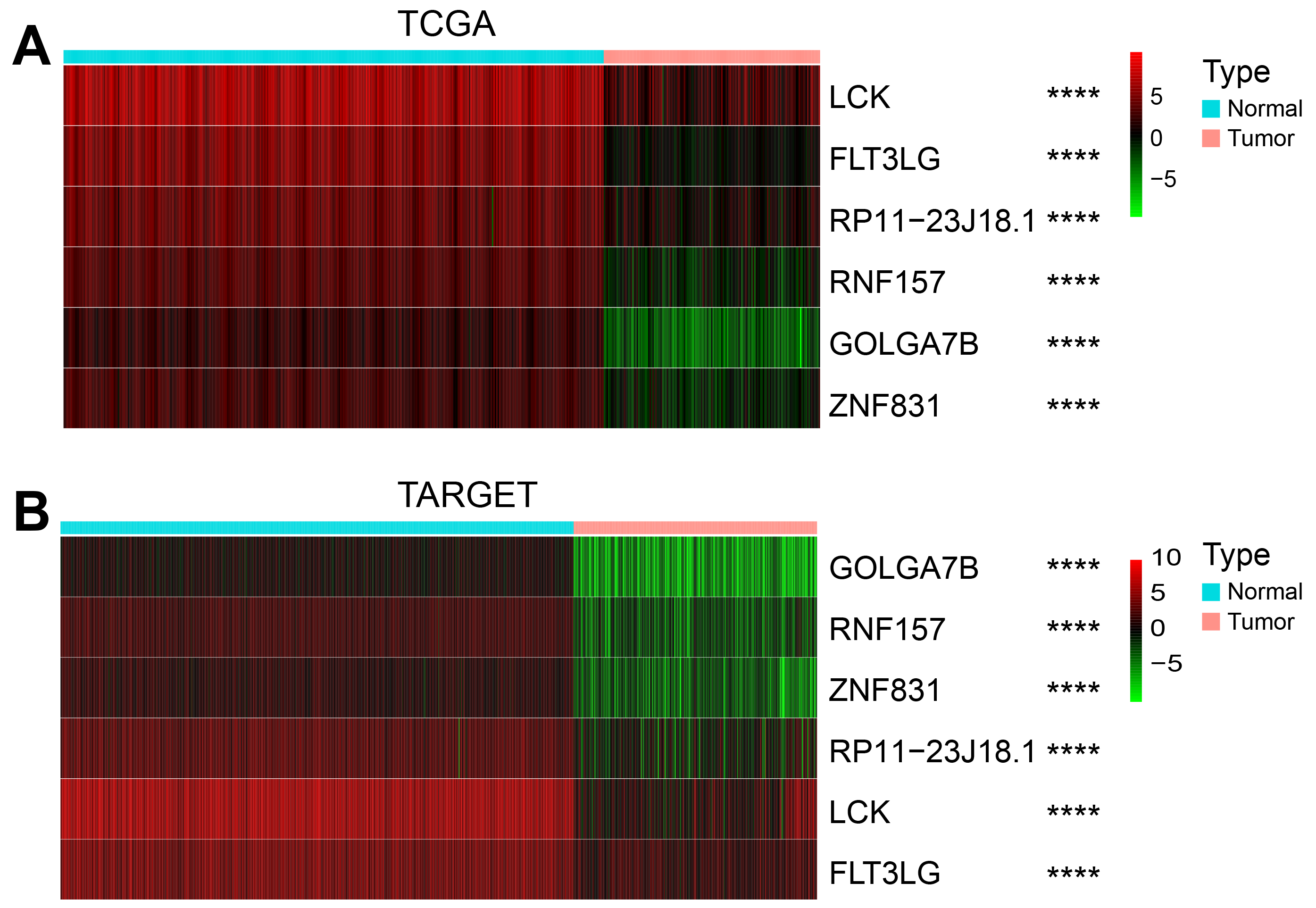

Supplement: Supplementary file 5 [file Image_5.tif]

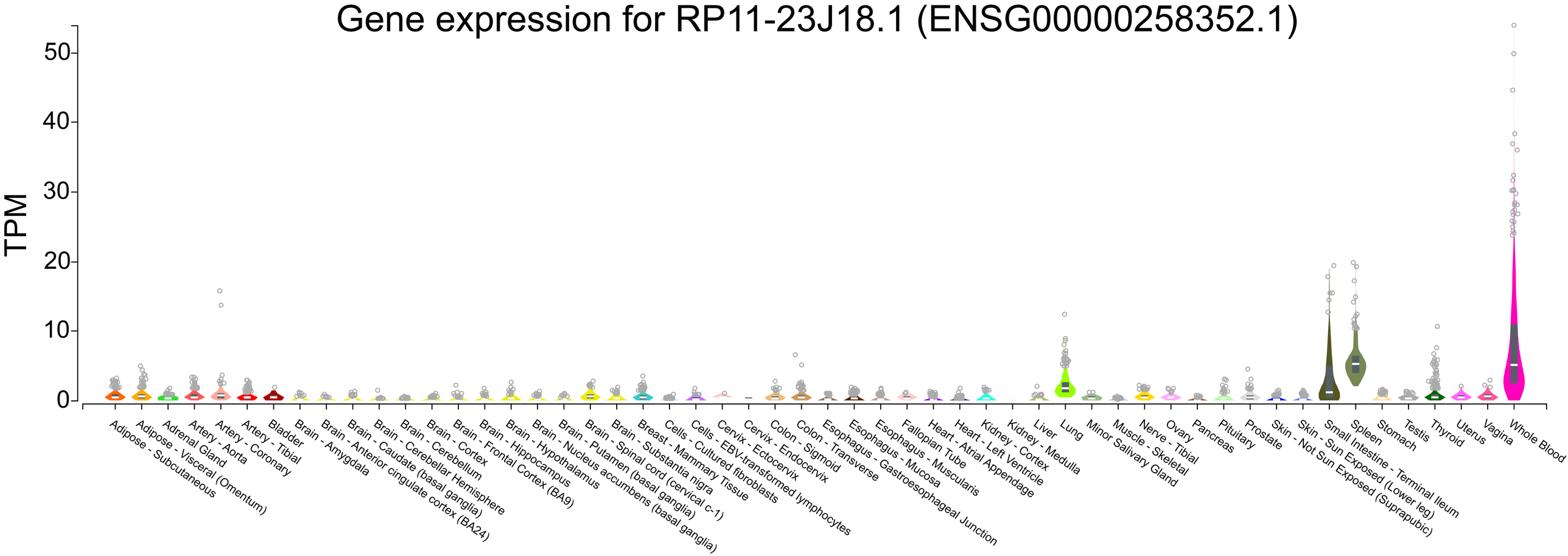

Supplement: Supplementary file 6 [file Image_6.tif]

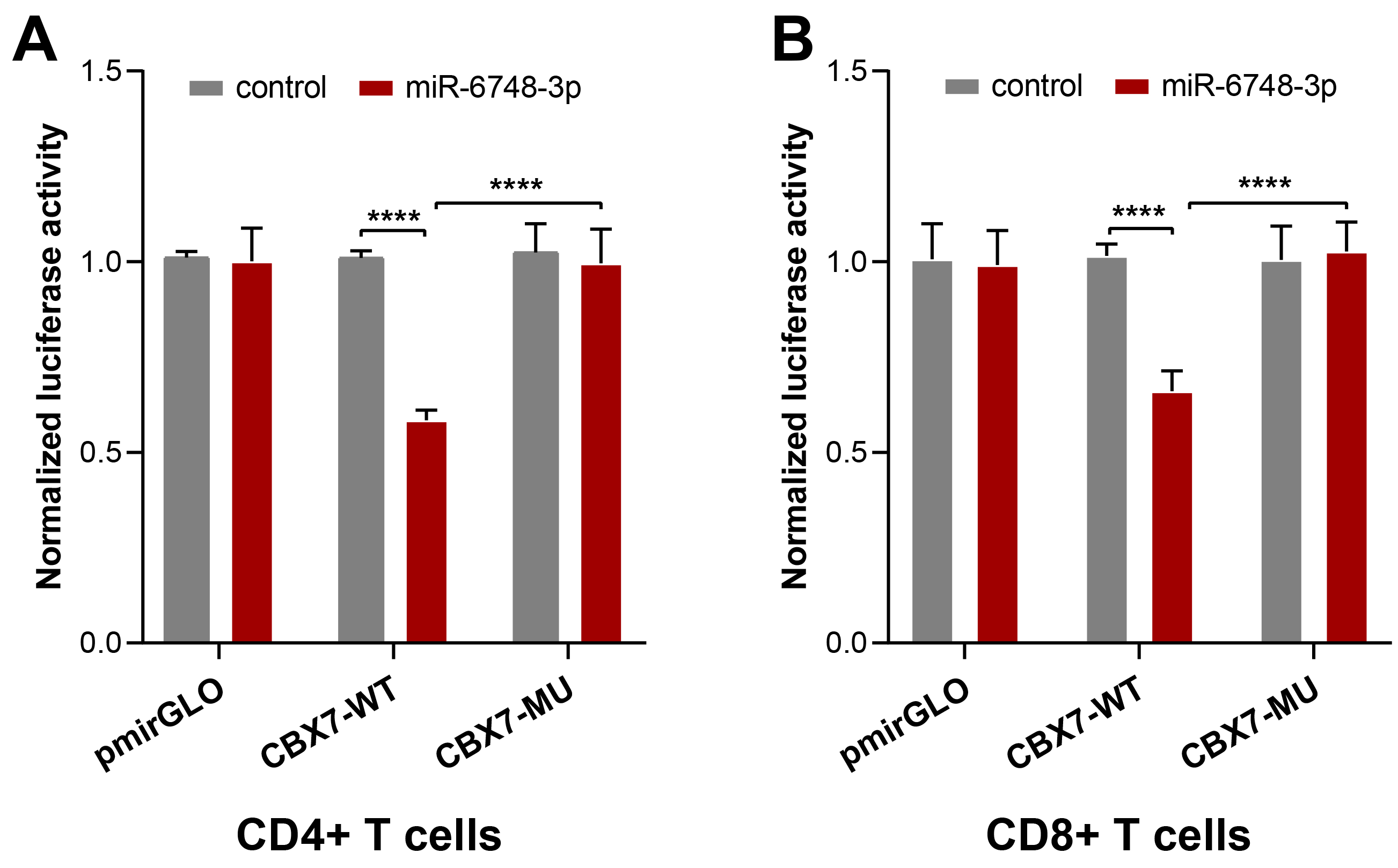

Supplement: Supplementary file 7 [file Image_7.tif]
