# Supplementary material for: FLT3LG and IFITM3P6 consolidate T cell activity in the bone marrow microenvironment and are prognostic factors in acute myelocytic leukemia
Source: Front Immunol. 2022 Aug 23;13:980911. doi: 10.3389/fimmu.2022.980911 (PMC9445253; doi:10.3389/fimmu.2022.980911)
Supplement: Supplementary file 9 [file Table_2.docx]

**Supplementary Table 2**

**• Primers for qRT-PCR**

| **Gene** | **Primers** |
| --- | --- |
| GAPDH (human) | Forward 5'-GAACGGGAAGCTCACTGG-3' |
|  | Reverse 5'-GCCTGCTTCACCACCTTCT-3' |
| U6 (human) | Forward 5'-CTCGCTTCGGCAGCACA-3' |
|  | Reverse 5'-AACGCTTCACGAATTTGCGT-3' |
| IFITM3P6 (human) | Forward 5'-TCTTCACTCCTGCCAACA-3' |
|  | Reverse 5'-AATAGGGACCAGACGACAT-3' |
| FLT3LG (human) | Forward 5'-GGCCCCTCTCATTCCCT-3' |
|  | Reverse 5'-GTGCTGGATTCTGGCGTT-3' |
| CBX7 (human) | Forward 5'-CGACATCACCGCCAACT-3' |
|  | Reverse 5'-GAAGTCCCACCCCAAGC-3' |
| CD40 (rat) | Forward 5'-ATACCCTCTGTGGTTTCCAGC-3'  Reverse 5'-TCCTTTGGTTTGACCACC-3' |
| GAPDH (rat) | Forward 5'-AGTGCCAGCCTCGTCTCATA-3' |
|  | Reverse 5'-TGAACTTGCCGTGGGTAGAG-3' |

**• Target sequence for overexpression of IFITM3P6**

ATGAACCACACTGTCCAAACCCTCTTCACTCCTGCCAACACCGGCCGCTCCACCAACCATGAGATGCTCAAGGAG

AAGCATGAGGTGGCTGTGCTGGGGGCACCCCACAACCCTGTGCCTCCAGCGTTCACCATGATCCACATCTGCAGT

GAGACCTCCGTGCCCGACCATGTCGTCTGGTCCCTATTCAACACCCTCTTCAAGAATTCCTGCTGCCCGGACTTCA

TAGCATTCATCTACTCTGTGAAGTCTAGGACAGGAAGTCTATGGACAGGAAGATGGTTGGTGACCTGACTGGGGC

CCAGGCCTGTGTCTCCACTGCCAAGTGCCTGAACATCTGGGCCCTGGCTCTGGGCATCCTCCTGACCATTCTGCTC

ATCATCATCTCAGTGCTGATCTTCCAA

**• Target sequences for downregulation of IFITM3P6**

sh-IFITM3P6-1: GGAAGTCTATGGACAGGAAGA (in priority)

sh-IFITM3P6-2: GCCCGGACTTCATAGCATTCA
